# Narrative Ability in Swedish Children Treated for Posterior Fossa Tumours: Macro‐ and Microstructural Performance Before and 1–4 Weeks After Surgery

**DOI:** 10.1111/1460-6984.70248

**Published:** 2026-04-24

**Authors:** K. Persson, D. Boeg Thomsen, K. Andersson, I. Tiberg, C. Castor, J. Grønbæk, R. Mathiasen, Å. Fyrberg

**Affiliations:** ^1^ Department of Health Sciences Lund University Lund Sweden; ^2^ Department of Nordic Studies and Linguistics University of Copenhagen Copenhagen Denmark; ^3^ Logopedics, Phoniatrics and Audiology, Department of Clinical Sciences Lund Lund University Lund Sweden; ^4^ Department of Paediatric and Adolescent Medicine Copenhagen University Hospital Rigshospitalet Copenhagen Denmark; ^5^ Department of Clinical Medicine Copenhagen University Copenhagen Denmark; ^6^ Child Neurology Assessment Unit, NUMO Sahlgrenska University Hospital Gothenburg Sweden

**Keywords:** cerebellar mutism syndrome (CMS), narrative ability, macrostructure, microstructure, posterior fossa tumour (PFT), speech, language and communication disorders

## Abstract

**Background:**

Posterior fossa tumours (PFT), among the most common childhood brain tumours, place children at risk of speech, language, and communication difficulties, often described in relation to cerebellar mutism syndrome (CMS) but also seen in children without mutism. Narrative ability refers to the capacity to understand, create, and share stories. Narratives can be analysed at the macrostructural level, capturing structure and content, and the microstructural level, reflecting, for example, morphosyntax and lexicon. Narrative ability has not previously been investigated in children with PFT at both levels, including pre‐ and postoperative data, or in relation to CMS or dysarthria.

**Aims:**

The aims were to investigate narrative ability at macrostructural and microstructural levels in children with PFT compared with typically developing (TD) peers, to investigate pre‐ to postoperative changes at both levels in children with PFT, and to examine relations between macro‐ and microstructure and the presence of CMS and dysarthria.

**Methods:**

A story‐generation task from the Expression, Reception and Recall of Narrative Instrument (ERRNI) was administered pre‐ and postoperatively, in the early postoperative phase (1–4 weeks after surgery). Macrostructure was analysed using ideascore, and microstructure using mean length of utterance in words (MLUw), grammaticality (GY), subordination index (SI), lexical diversity (MATTR), and words per minute (WPM). Group comparisons and associations with CMS and dysarthria were analysed using linear regressions, and changes over time with mixed‐effects models.

**Results:**

Children with PFT scored significantly lower than TD peers on the macrostructural measure ideascore and on several microstructural measures, including MLUw, GY, and WPM, both pre‐ and postoperatively (all *p *< 0.05). SI and MATTR did not differ between groups. Difficulties were most pronounced among older children. No significant pre‐ to postoperative changes were found, although individual variability was evident. The small group of children with CMS did not differ significantly from other children with PFT, while those with postoperative dysarthria showed a decline in both macro‐ and microstructure (*p *< 0.05).

**Conclusions:**

Children with PFT showed pre‐ and postoperative difficulties at macro‐ and microstructural levels, producing narratives with fewer story elements, shorter utterances, and more grammatical errors compared with TD peers. The age‐related differences suggest that narrative difficulties become more prominent as language and cognitive demands increase. The findings underline the clinical importance of including narrative tasks encompassing macro‐ and microstructural aspects, together with motor‐speech evaluation, in assessment and follow‐up of children treated for PFT to guide appropriate interventions.

**WHAT THIS STUDY ADDS:**

*What is already known on this subject*

- Previous studies have described speech, language and/or communication difficulties following posterior fossa tumour surgery, particularly in association with cerebellar mutism syndrome (CMS). One aspect of language that may be affected is narrative ability, which can be analysed at two levels: the macrostructural level (content) and the microstructural level (form). Still, little is known about narrative ability in children with posterior fossa tumours (PFT), although a small study based on postoperative data has shown microstructural difficulties in children undergoing PFT surgery.

*What this paper adds to existing knowledge*

- Our study adds new knowledge about language profiles in children with PFT, showing that they have narrative difficulties at macro‐ as well as at microstructural levels, present both pre‐ and postoperatively. These difficulties are most evident among older children and show individual variability. We did not find more pronounced difficulties in the small group of children with CMS compared with the other children undergoing PFT surgery.

*What are the potential or actual clinical implications of this work?*

- The findings underline the importance of including narrative assessment in the evaluation and follow‐up of children treated for PFT. Assessing macro‐ and microstructural aspects together with motor‐speech evaluation can help identify children in need of interventions. Regular follow‐up assessments are essential, as narrative difficulties may become more apparent as language and cognitive demands increase.

**Abbreviations**
ABI: acquired brain injury
ADHD: attention‐deficit/hyperactivity disorder
CI: confidence interval
CMS: cerebellar mutism syndrome
CNS: central nervous system
C‐units: communication units
DLD: developmental language disorder
ERRNI: expression, reception and recall of narrative instrument
GY: grammaticality
ICC: intraclass correlation coefficient
IRR: inter‐rater reliability
MATTR: moving average type–token ratio
MLUw: mean length of utterance in words
PFT: posterior fossa tumour
SALT: systematic analysis of language transcripts
SD: standard deviation
SI: subordination index
TD: typically developing
TTR: type–token ratio
WPM: words per minute

## 1 Introduction

Narrative ability refers to the capacity to understand, create, and share stories, requiring the coordination of multiple language and cognitive processes (Bishop 2004). It plays a central role in the development of language comprehension and production and is a key predictor of academic performance, particularly literacy and reading achievement (Babayiğit et al. 2021; Griffin et al. 2004; Reese et al. 2011). Beyond academics, narrative ability is essential for social interaction, sharing personal experiences, and engaging in humour, which form the foundation for social acceptance and group belonging (Berman 1988). Narrative ability develops throughout childhood and adolescence and continues to develop across the life span. After the age of five, development is largely characterised by refinement and increasing complexity: narratives become longer, more elaborated, and marked by advances in syntactic complexity, coherence, and the ability to adapt stories for a listener's understanding. Within this process, preschool narratives remain largely event‐driven with limited coherence and minimal temporal or causal connections (Kupersmitt and Nicoladis 2021). As children enter middle childhood, they increasingly use story grammar elements, such as setting and causal sequencing, accompanied by gains in syntactic complexity (Nippold et al. 2014). By adolescence, narratives demonstrate greater coherence, abstraction, and perspective‐taking, reflecting advances in language processing and higher‐order cognitive functions (Kupersmitt and Nicoladis 2021) as well as narrative experience.

To capture this complexity, narratives can be analysed at two levels: macrostructural and microstructural level (Berman 1988). The macrostructural level refers to the global framework and organisation of a narrative, capturing how coherent and well‐structured the story is. This level encompasses elements such as logical sequencing, thematic continuity, and the inclusion of components like setting, initiating events, and resolutions, which together reflect the overarching clarity and completeness of the narrative. Macrostructure can be evaluated through different approaches. One such approach is the measure ideascore, which reflects how effectively a child conveys the main ideas of a narrative (Bishop 2004). The microstructural level refers to the language details within the narrative, such as sentence structure, grammatical accuracy, and vocabulary use. This level can be evaluated using a range of microstructural language measures. Mean length of utterance in words (MLUw) provides an indicator of sentence length and is often used as a rough measure of syntactic complexity. However, utterance length alone is not a sufficient index of syntactic complexity, as longer utterances may simply reflect concatenation rather than more complex constructions (Frizelle et al. 2018). To capture syntactic complexity more precisely, measures of clause subordination are applied, as these indicate the child's ability to produce embedded structures. Grammaticality reflects morphosyntactic accuracy (Miller et al. 2016). Lexical diversity captures the range of vocabulary used within the narrative, indicating both the breadth and flexibility of the child's lexical repertoire (Malvern et al. 2004). Fluency‐related aspects such as pausing and speech rate reflect the ease of speech production (Berman 1988; Kapantzoglou et al. 2017).

Macro‐ and microstructural levels together provide a comprehensive view of narrative production, making narrative analysis a valuable approach for assessing both language and communicative abilities in children. Studies of children with developmental language disorder (DLD) report difficulties at both levels, including shorter and less coherent narratives, reduced grammatical accuracy, and fewer story grammar elements as well as limited use of internal state terms compared with typically developing (TD) peers (Andreou and Lemoni 2025; Reilly et al. 2004; Winters et al. 2022). Similar narrative difficulties have also been described in other paediatric groups, such as in children with autism spectrum disorder and foetal growth restriction (Partanen et al. 2020; Volden et al. 2017). Narrative analyses are also valuable for children with acquired brain injury, including those treated for brain tumours, since they enable examination of multiple language measures and can be carried out efficiently. In a study by Docking et al. (2016), narratives were analysed in children after brain tumour treatment. While no significant group differences were found compared with age‐ and gender‐matched peers, many individual children performed below the comparison group on measures of lexical diversity, total number of utterances, mean length of utterance, grammatical accuracy, and overall narrative structure (Docking et al. 2016).

Tumours in the posterior fossa are the most common paediatric brain tumours, accounting for nearly half of all central nervous system (CNS) tumours in children (Ostrom et al. 2023). Surgical resection is essential and advances in treatment have improved survival rates for children with CNS tumours, but many survivors face neurological and functional complications (Cámara et al. 2020; Catsman‐Berrevoets and Aarsen 2010; Di Rocco et al. 2010). Cerebellar mutism syndrome (CMS), one of the most severe complications, is characterised by mutism or severely reduced speech, along with emotional lability and other neurological deficits (Gudrunardottir et al. 2016; Grønbæk et al. 2021, 2023). While CMS often improves over time, many children experience persistent speech and language disorders that impact communication and academic performance (Catsman‐Berrevoets and Aarsen 2010; Svaldi et al. 2023). The language disorders comprise both comprehension and production, with difficulties reported in morphosyntax and lexical semantics (Di Rocco et al. 2011; Svaldi et al. 2023; Riva and Giorgi 2000). While these findings provide valuable insights into language disorders in this population, many studies rely on standardized neuropsychological assessments, which do not capture the essential aspects of language (Di Rocco et al. 2010; Catsman‐Berrevoets and Aarsen 2010; Cámara et al. 2020). A further uncertainty pertains to the role of the tumour versus the role of surgery in causing language disorders. The majority of studies only analyse postoperative data, making it impossible to separate the impact of tumour and surgery, but recent findings based on language assessments indicate that children with PFT already show word‐finding difficulties before surgery (Persson et al. 2023), indicating a negative impact of the tumour itself. Moreover, postoperative language outcomes vary between individuals (Persson et al. 2025), indicating that surgery may have different effects on different children. One potential factor affecting individual language outcomes after surgery could be whether the child has experienced a phase of CMS. While studies have often focused on the language disorders that may follow CMS, language disorders following posterior fossa tumour (PFT) surgery also sometimes occur in children without CMS, emphasising the broader impact of these tumours and their treatment on language outcomes (Cámara et al. 2020; De Smet et al. 2009). Moreover, a recent study by Svaldi et al. (2023) identified deficits at the microstructural level in spontaneous speech among children following PFT. These difficulties were evident in decreased lexical diversity, morphosyntactic deficits together with reduced MLUw and difficulties with verb transitivity. These deficits were observed in both children with and without CMS, although morphosyntactic and semantic difficulties appeared more prominent in the CMS group (Svaldi et al. 2023). Yet, this study was limited to postoperative data from a small sample and did not examine macrostructural narrative ability, and it remains unclear whether children with CMS exhibit a different or more pronounced pattern of narrative difficulties compared with those without CMS. Another potential factor affecting individual language outcomes after surgery is the presence or absence of dysarthria. Postoperatively, motor speech disorder is common, typically dysarthria, characterised by slow rate, monotonous tone, and ataxic speech (De Smet et al. 2007; Van Mourik et al. 1998). Dysarthria concerns the motor execution of speech and is not expected to directly affect language ability, but it may influence a child's language production since the increased effort required for speech can make it more difficult to produce coherent, long and fluent utterances.

Previous research on language deficits in children with PFT has mainly focused on the postoperative period (Catsman‐Berrevoets and Aarsen 2010; Grønbæk et al. 2021). Without a preoperative assessment, it is difficult to determine whether difficulties observed after surgery reflect a deterioration associated with the surgical intervention or are attributable to the tumour itself. Furthermore, individual variability has been reported, with some children improving and others declining following surgery (Di Rocco et al. 2011; Persson et al. 2025). Given this heterogeneity, it is essential to include both pre‐ and postoperative assessments and to examine changes over time.

Therefore, in the current study, we aimed to:

1. Investigate narrative macrostructure and microstructure in children with posterior fossa tumours, assessed pre‐ and postoperatively, compared with typically developing peers.
2. Investigate change in narrative macrostructure and microstructure between pre‐ and postoperative assessment in children with posterior fossa tumours.
3. Investigate change in narrative macrostructure and microstructure between pre‐ and postoperative assessment in children with posterior fossa tumours in relation to the presence of cerebellar mutism syndrome or dysarthria.

## 2 Methods

### 2.1 Study Design and Setting

This was a prospective comparative observational cohort study including children undergoing PFT surgery and a comparison group of typically developing (TD) children. Data for the PFT children were derived from the Swedish part of the Nordic‐European CMS study, described elsewhere (Wibroe et al. 2017), which had collected these data at paediatric oncology centres in Sweden. A normative comparison group of typically developing (TD) children was recruited from educational settings in southern Sweden. The Swedish part of the Nordic‐European CMS study was approved by the Swedish Ethical Review Board (reference 2014/3:8), and a separate ethical approval was obtained for the TD comparison group (reference 2024‐05489‐01).

### 2.2 Participants

Between August 2014 and May 2024, 177 Swedish children under the age of 18 years were included in the Nordic‐European CMS study. For the present study, children from this cohort were considered for inclusion if they had available audio‐recorded narrative assessments pre‐ and/or postoperatively. To minimise the influence of potential confounding factors, the following exclusion criteria were applied for the present study: additional diagnoses such as neurodevelopmental disorders (e.g., autism and ADHD), previously reported speech and language disorders and previous tumour surgery. In some cases, information regarding earlier speech or language difficulties was reported only as the presence of prior difficulties, with further details provided in free‐text fields. When such information indicated that the reported difficulties were tumour‐related rather than developmental in nature, the children were retained in the study; otherwise, they were excluded. Children with a multilingual background were also excluded, as information on length and quality of exposure to the Swedish language was not available. Children younger than five years were also excluded, as narrative ability becomes more stable and structured from around the age of five. Recordings with missing or invalid data due to experimenter error were also discarded. The final sample included 44 children with valid narrative data either pre‐ and/or postoperatively. Of these, 32 had preoperative data, 32 postoperative data (comprising 64 narratives in total), and 20 had both pre‐ and postoperative data.

Children included for the collection of normative data were recruited from schools and preschools in southern Sweden between January and April 2024. Initially, 151 children aged between 5 and 17 years were enrolled. The same exclusion criteria were applied as for the tumour group: neurodevelopmental disorders (e.g., autism and ADHD), previously reported speech and language disorders, neurological conditions, or multilingualism. Recordings with missing or invalid data due to experimenter error were also discarded. The final control group comprised 118 children.

**FIGURE 1:**
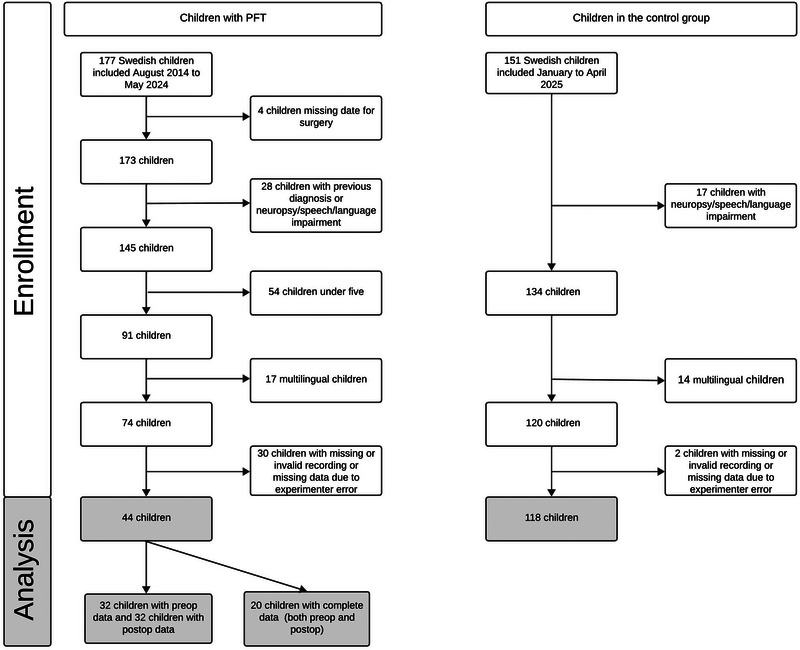
The inclusion process and the children included in each analysis.

The distribution of age groups (5–7, 8–12, ≥13 years) did not differ significantly between children with PFT and TD peers (*χ* ^2^(2, *n =* 162) = 0.74, *p =* 0.69), with the majority of children falling into the 8–12‐year range. The sex ratio was balanced in the group of children with PFT, while girls were slightly more represented among the children with TD; however, the difference in sex distribution was not statistically significant (*χ* ^2^(1, *n =* 162) = 1.23, *p =* 0.27). Figure 1 presents the inclusion process and the number of participants included in each analysis.

### 2.3 Materials

To elicit narratives, the Expression, Reception, and Recall of Narrative Instrument (ERRNI) was used (Bishop 2004). The original British version of the ERRNI has demonstrated good interrater and test–retest reliability as well as construct and concurrent validity (Bishop 2004). The Swedish version of the ERRNI was developed and evaluated in a master thesis in 2009 (Gustafsson et al. 2009). In that study, interrater reliability was reported to be high, and children's performance on the Swedish ERRNI followed the same age‐related patterns as those observed in the original British version, supporting its suitability for use with Swedish‐speaking children. The assessment in this study involved the story generation part of the Fish Story task, which includes a set of 15 pictures. Using these sequential images, children were supported in generating an independent narrative.

### 2.4 Data Collection

Demographic and medical data as well as audio‐recorded narrative files for the children with PFT were obtained from the Nordic‐European CMS study. The demographic and clinical information was reported prospectively via study forms completed by physicians. Dysarthria was reported using a four‐point scale ranging from normal speech to absent/unintelligible speech. Tumour location, documented after surgery, could include multiple sites within the posterior fossa; left cerebellar hemisphere, right cerebellar hemisphere, cerebellar vermis, fourth ventricle, and/or brainstem involvement. Tumour histology was categorised as pilocytic astrocytoma, medulloblastoma, ependymoma, atypical teratoid rhabdoid tumour, or other. Hydrocephalus was reported as either absent or present in the study forms. Mutism was defined as “absence of speech with no production of words or short sentences, though crying or whining may still occur in some children.” Reduced speech was defined as “severely limited speech production, restricted to single words or short sentences, only elicited after vigorous stimulation.” The presence of mutism or reduced speech was documented, and when present, the duration (number of days) was noted. As children with unresolved mutism could not participate in the postoperative language assessment, no postoperative language samples are available for these cases. However, if mutism resolved within the 1–4‐week postoperative window, the child was included once speech production was regained; children with reduced speech were included provided they were able to participate in the assessment.

Within the Nordic‐European CMS study, the narrative assessment using Fish Story was administered in a quiet setting at the paediatric oncology centres by a physician, nurse, or speech‐language therapist. The administration followed written instructions. Children were instructed to carefully examine the sequence of pictures before beginning their narrative. During the narrative task, minimal encouragement was provided by the examiner, who used neutral prompts such as “What happened next?” or “Tell me more”. Preoperative assessments were conducted as close as possible to the date of surgery, typically within the last few days before the procedure. Postoperative assessments were carried out 1–4 weeks after surgery, while the children were still hospitalised for postoperative care. The narrative assessments were audio‐recorded, and the files were stored in the Nordic‐European CMS database.

For the children with TD recruited for the current study as age‐matched controls the demographic data, including birthdate, sex, language background, multilingualism, and any reported speech, language, or other diagnoses, were collected through parent‐reported questionnaires. The narrative assessment using Fish Story was administered in a quiet setting at the children's schools by a speech‐language therapist or supervised speech‐language therapist students. The test administration followed the same written instructions as within the Nordic‐European CMS study. The narrative assessments were audio‐recorded and the files were stored.

The subsequent steps of transcription, assessment, and analysis, based on the audio files, were conducted by the first author and applied in the same way to data from both the PFT and the TD groups.

### 2.5 Orthographic Transcription

The transcription process began with an initial rough transcription of the recordings using the @Train software (Haberl et al. 2024). The recordings were then transcribed orthographically with the use of headphones, in a quiet environment. All narratives were transcribed by a single transcriber, the first author, to ensure consistency across the data.

### 2.6 Assessment of Macrostructure

To assess the ideascore, all narratives were evaluated according to the ERRNI manual (Bishop 2004). Each of the 24 pre‐determined story items was assigned a score ranging from 0 to 2 points: 2 points were awarded for a fully correct and detailed expression of the idea, 1 point for partial or vague information, and 0 points for omitted or incorrectly expressed items. The total ideascore was calculated by summing the scores for all items in each narrative.

### 2.7 Assessment of Microstructure

The narratives were orthographically transcribed and coded following the Systematic Analysis of Language Transcripts (SALT) guidelines (Miller et al. 2016). Sentence length was measured using MLUw, calculated by dividing the total number of words by the total number of complete and intelligible communication units (C‐units; a main clause with all subordinate clauses). Grammaticality (GY) was calculated as the percentage of utterances containing grammatical errors, by dividing the number of incorrect C‐units by the total number of C‐units. Thus, higher values indicate lower grammatical accuracy. Syntactic complexity was represented by the subordination index (SI), calculated by dividing the total number of clauses, including both main and subordinate clauses, by the number of C‐units; a higher index reflects more advanced syntactic complexity. Lexical diversity was assessed using the moving average type–token ratio (MATTR), which calculates the average type–token ratio from multiple consecutive subsamples of a transcript. In contrast to the traditional type–token ratio (TTR), MATTR is not sensitive to sample length, making it more suitable for comparing narratives of different lengths. The window size was set to 21 tokens, corresponding to the length of the shortest sample included (22 tokens). Fluency‐related aspects were captured by speech rate, measured as words per minute (WPM), calculated by dividing the total number of words produced by the duration of the sample in minutes. All calculations were performed using SALT 2018 Research Version.

### 2.8 Agreement

To ensure reliability of assessment, inter‐rater reliability (IRR) was calculated for 10% of the narratives. Inter‐rater reliability for macrostructural analysis was assessed by the first and last authors, yielding a mean Cohen's Kappa of 0.82, indicating a high level of agreement. Inter‐rater reliability for microstructural analysis was assessed by the first and third authors, with reliability calculated using intraclass correlation coefficients (ICC). A mean ICC of 0.86 was obtained, demonstrating strong consistency between raters. Intra‐rater reliability was evaluated for both the macrostructural (ideascore) and microstructural measures, as the same rater assessed all narratives. To achieve this, 10% of the narratives were randomly selected. Initially, 10 narratives were scored, followed by a washout period of 3 weeks to minimize recall bias. After this period, the same narratives were re‐evaluated. Intra‐rater reliability for the ideascore was assessed using Cohen's Kappa, with a mean value of 0.91, indicating excellent consistency. For the microstructural measures, intra‐rater reliability was evaluated using the intraclass correlation coefficient (ICC), which resulted in a mean ICC of 0.97, demonstrating a very high level of agreement across repeated evaluations.

### 2.9 Statistical Analysis

Demographic and clinical characteristics were presented descriptively. To compare pre‐ and postoperative macro‐ and microstructure in children with PFT to children with TD, linear regression models were fitted for each outcome. The dependent variables were ideascore, MLUw, GY, SI, MATTR, and WPM, and the independent variable was group (TD children, preoperative PFT assessments, and postoperative PFT assessments) with TD as the reference category. All models included age and sex as control variables. To complement the group‐level findings, we examined the extent of the difficulties at the individual level. For each age group (5–7, 8–12, ≥13 years), the TD mean and standard deviation were calculated, and each PFT observation was classified as within one SD of the TD mean, 1–2 SD below, or more than 2 SD below. For GY, where higher values indicate poorer accuracy, deviations were defined in the opposite direction. SDs are commonly applied in clinical assessment where scores 1–2 SD below the mean indicate moderate weakness and scores more than 2 SD below indicate severe difficulties (Semel et al. 2013).

To investigate change in macro‐ and microstructure between pre‐ and postoperative assessment in children with PFT, linear mixed‐effects models were fitted with each outcome (ideascore, MLUw, GY, SI, MATTR, WPM) as the dependent variable. All available observations from children with PFT were included, with participant as a random intercept. Fixed effects were specified for timepoint (pre vs. post), with age and sex included as control variables.

To investigate change in macrostructure and microstructure in children with PFT in relation to the presence of cerebellar mutism syndrome (CMS) or dysarthria, analyses were restricted to children with complete pre‐ and postoperative data. For each outcome (ideascore, MLUw, GY, SI, MATTR, WPM), individual change scores (post—pre) were calculated. Children were classified according to the presence of postoperative cerebellar mutism syndrome (CMS) or dysarthria, which were analysed in separate models, with children without the respective condition serving as the reference group. Linear regression models were fitted with change for each outcome as the dependent variable, group as independent variable and all models adjusted for age and sex.

For all change analyses, standardised regression coefficients (*β*_std_) were additionally calculated to quantify effect size in standard deviation units, thereby facilitating comparison across outcomes with different measurement scales. All analyses were performed in RStudio (version 2025.09.0+387).

## 3 Results

The sample consisted of 44 children with posterior fossa tumours and 118 typically developing (TD) peers. Among the children with PFT, 32 contributed preoperative data, 32 postoperative data, and 20 had complete data at both timepoints. Of the children with PFT, 12 developed postoperative CMS symptoms, three children developed mutism, aged 7:5, 11:0, and 12:5 years, and nine children had reduced speech, with ages ranging from 5.6 to 15.8 years. Duration data were available for one child with mutism, with a duration of 2 days, and for five of the nine children with reduced speech, with individual durations of 2–4 days. Dysarthria was documented in 3 children preoperatively and 5 children postoperatively. Demographic data for all children with PFT, for the subsets with preoperative, postoperative, and complete data, and for all TD children are presented in Table 1.

**TABLE 1:** Demographic and clinical characteristics of children with posterior fossa tumours (PFT) and typically developing (TD) peers.

	All children undergoing PFT surgery (*n =* 44)	Children with preoperative data (*n =* 32)	Children with postoperative data (*n =* 32)	Children with complete pre‐ and postoperative data (*n =* 20)	All children with typical development (*n =* 118)
Age (median, Q1, Q3)	9:11, 7:10, 12:3	10:7, 8:2, 12:3	9:11, 8:1, 12:3	11:0, 8:4, 12:11	9:11, 8:1, 12:1
Age group, n (%)					
5–7	12 (27.3)	8 (25)	8 (25)	4 (20)	26 (22)
8–12	24 (54.5)	18 (56.2)	17 (53.1)	11 (55)	73 (61.9)
≥13	8 (18)	6 (18.8)	7 (21.9)	5 (25)	19 (16.1)
Sex, *n* (%)					
Girls	20 (45.5)	15 (46.9)	16 (50)	11 (55)	67 (56.8)
Boys	24 (54.5)	17 (53.1)	16 (50)	9 (45)	51 (43.2)
CMS, *n* (%)					
Mute	3 (7.5)	3 (10.7)	1 (3.4)	1 (5.9)	—
Reduced speech	9 (22.5)	6 (21.4)	6 (20.7)	3 (17.6)	—
Habitual speech	28 (70)	19 (67.9)	22 (75.9)	13 (76.5)	—
Unknown	4	4	3	3	—
Tumour location, *n* (%)					
LCH	3 (7)	3 (9.7)	1 (3.1)	1 (5)	—
LCH + FV	2 (4.7)	1 (3.2)	1 (3.1)	—	—
RCH	5 (11.6)	2 (6.5)	5 (15.6)	2 (10)	—
RCH + LCH	2 (4.7)	2 (6.5)	2 (6.2)	2 (10)	—
RCH + VR	1 (2.3)	1 (3.2)	1 (3.1)	1 (5)	—
VR	6 (14)	3 (9.7)	5 (15.6)	2 (10)	—
VR + FV	2 (4.7)	2 (6.5)	1 (3.1)	1 (5)	—
BS	3 (7)	3 (9.1)	1 (3.1)	1 (5)	—
BS+FV/VR	2 (4.7)	2 (6.5)	2 (6.2)	2 (10)	—
FV	7 (16.3)	4 (12.9)	6 (18.8)	3 (15)	—
≥3 locations	10 (23.3)	8 (25.8)	7 (21.9)	5 (25)	—
Unknown	1	1	0	0	—
Tumour histology, n (%)					
Pilocytic astrocytoma	19 (50)	14 (51.9)	14 (48.3)	9 (50)	—
Medulloblastoma	14 (36.8)	11 (40.7)	10 (34.5)	7 (38.9)	—
Ependymoma	2 (5.3)	1 (3.7)	2 (6.9)	1 (5.6)	—
Other	3 (7.9)	1 (3.7)	3 (10—3)	1 (5.6)	—
Unknown	6	5	3	2	—
Dysarthria, pre‐ postop *n* (%)					
Present		2 (6.9)	4 (14.8)	1 (5.3), 3 (15.8)	—
Absent		27 (93.1)	23 (85.2)	18 (94.7), 16 (84.2)	—
Unknown		3	5	1	—
Hydrocephalus, pre‐ postop *n* (%)					
Present		14 (60.9)	1 (5)	12 (80), 1 (6.7)	—
Absent		9 (39.1)	19 (95)	3 (20), 14 (93.3)	—
Unknown		9	12	5.5	—

*Note*: Data are presented for all children with PFT, and for the subsets with preoperative, postoperative, and complete data, as well as for all TD children. Age is reported as median with first (Q1) and third quartiles (Q3).

Abbreviations: CMS, cerebellar mutism syndrome; LCH, left cerebellar hemisphere; RCH, right cerebellar hemisphere; BS, brainstem; VR, vermis; FV, fourth ventricle.

Other includes atypical teratiod rhabdoid tumour and other tumours.

### 3.1 Narrative Difficulties in Children With PFT Compared With TD Peers

On our measure of macrostructure, ideascore, children with PFT scored significantly lower than TD peers, both preoperatively (*β* = −4.82, 95% CI −7.14 to −2.50, *p *< 0.001) and postoperatively (*β* = −7.05, 95% CI −9.37 to −4.73, *p *< 0.001). For the microstructural outcomes, significant group differences were observed for MLUw, GY, and WPM. Preoperatively, children with PFT produced shorter MLUw (*β* = −0.82, 95% CI −1.50 to −0.14, *p *< 0.01), had more grammatical errors (*β* = +0.06, 95% CI 0.02 to 0.09, *p *< 0.01), and fewer WPM (*β* = −20.7, 95% CI −34.69 to −6.58, *p *< 0.001). Postoperatively, differences between children with PFT and TD peers were even larger for MLUw (*β* = −1.55, 95% CI −2.22 to −0.87, *p *< 0.001), while the difference for GY (*β* = +0.04, 95% CI 0.01 to 0.07, *p *< 0.05) and WPM (*β* = −18.3, 95% CI −32.39 to −4.32, p < 0.05) was of similar magnitude. No significant group differences were found for SI or MATTR at either timepoint. The models are presented in Table 2

**TABLE 2:** Linear model estimates comparing children with posterior fossa tumours (PFT; preoperative, postoperative) with typically developing (TD) peers for macrostructural (ideascore) and microstructural (MLUw, GY, SI, MATTR, WPM) outcomes, adjusted for age and sex.

	B (95% CI)	p‐value	Standardised *β* (95% CI)
**Ideascore**			
PFT preop	−4.82 (‐7.14 – ‐2.50)	**<0.001**	−0.64 (‐0.95 – ‐0.33)
PFT postop	−7.04 (‐9.37 – ‐ 4.73)	**<0.001**	−0.94 (‐1.25 – ‐0.63)
Age	1.11 (0.81 – 1.40)	**<0.001**	0.45 (0.33 – 0.56)
Sex: boys	−1.81 (‐3.57 – ‐ 0.06)	**<0.05**	−0.24 (‐0.48 – ‐0.01)
**MLUw**			
PFT preop	−0.82 (‐1.50 – ‐0.14)	**<0.01**	−0.39 (‐0.71 – ‐0.07)
PFT postop	−1.55 (‐2.22 – ‐0.87)	**<0.001**	−0.73 (‐1.05 – ‐0.41)
Age	0.35 (0.26 – 0.43)	**<0.001**	0.49 (0.37 – 0.62)
Sex: boys	−0.40 (‐0.99 – 0.08)	0.099	−0.20 (‐0.44 – 0.04)
**GY**			
PFT preop	0.06 (0.02 ‐ 0.09)	**<0.01**	0.64 (0.26 – 1.02)
PFT postop	0.04 (0.01 ‐ 0.07)	**<0.05**	0.40 (0.02 – 0.77)
Age	0.01 (‐0.08 – 0.01)	0.148	−0.11 (‐0.25 – 0.04)
Sex: boys	0.01 (‐0.01 – 0.04)	0.192	0.19 (‐0.10 – 0.48)
**SI**			
PFT preop	−0.06 (‐0.24 – 0.13)	0.549	−0.12 (‐0.50 – 0.27)
PFT postop	−0.10 (‐0.29 – 0.12)	0.261	−0.22 (‐0.60 – 0.17)
Age	0.04 (0.01 – 0.06)	**<0.01**	0.23 (0.09 – 0.38)
Sex: boys	−0.03 (‐0.16 – 0.12)	0.712	−0.05 (‐0.35 – 0.24)
**MATTR**			
PFT preop	0.00 (‐0.03 – 0.01)	0.771	−0.05 (‐0.42 – 0.31)
PFT postop	−0.01 (‐0.04 – 0.00)	0.173	−0.25 (‐0.61 – 0.11)
Age	0.005 (0.005 – 0.010)	**<0.01**	0.38 (0.25 – 0.52)
Sex: boys	0.00 (‐0.02 – 0.01)	0.292	−0.15 (‐0.42 – 0.13)
**WPM**			
PFT preop	−20.7 (‐34.69 – ‐6.58)	**<0.01**	−0.53 (‐0.88 – ‐0.17)
PFT postop	−18.3 (‐32.39 – ‐4.32)	**<0.05**	−0.47 (‐0.83 – ‐0.11)
Age	4.63 (2.86 – 6.40)	**<0.001**	0.35 (0.22 – 0.49)
Sex: boys	−3.63 (‐14.28 – 7.00)	0.500	−0.09 (‐0.36 – 0.18)

*Note*: Unstandardised (*β*) and standardised (*β*_std_) coefficients with 95% confidence intervals (CI) are reported. Bold values indicate statistically significant effects (p<.05).

Ideascore is narrative macrostructure score.

Abbreviations: GY, grammaticality (higher values = poorer accuracy); MATTR, moving average type–token ratio; MLUw, mean length of utterance in words; SI, subordination index; WPM, words per minute.

**FIGURE 2:**
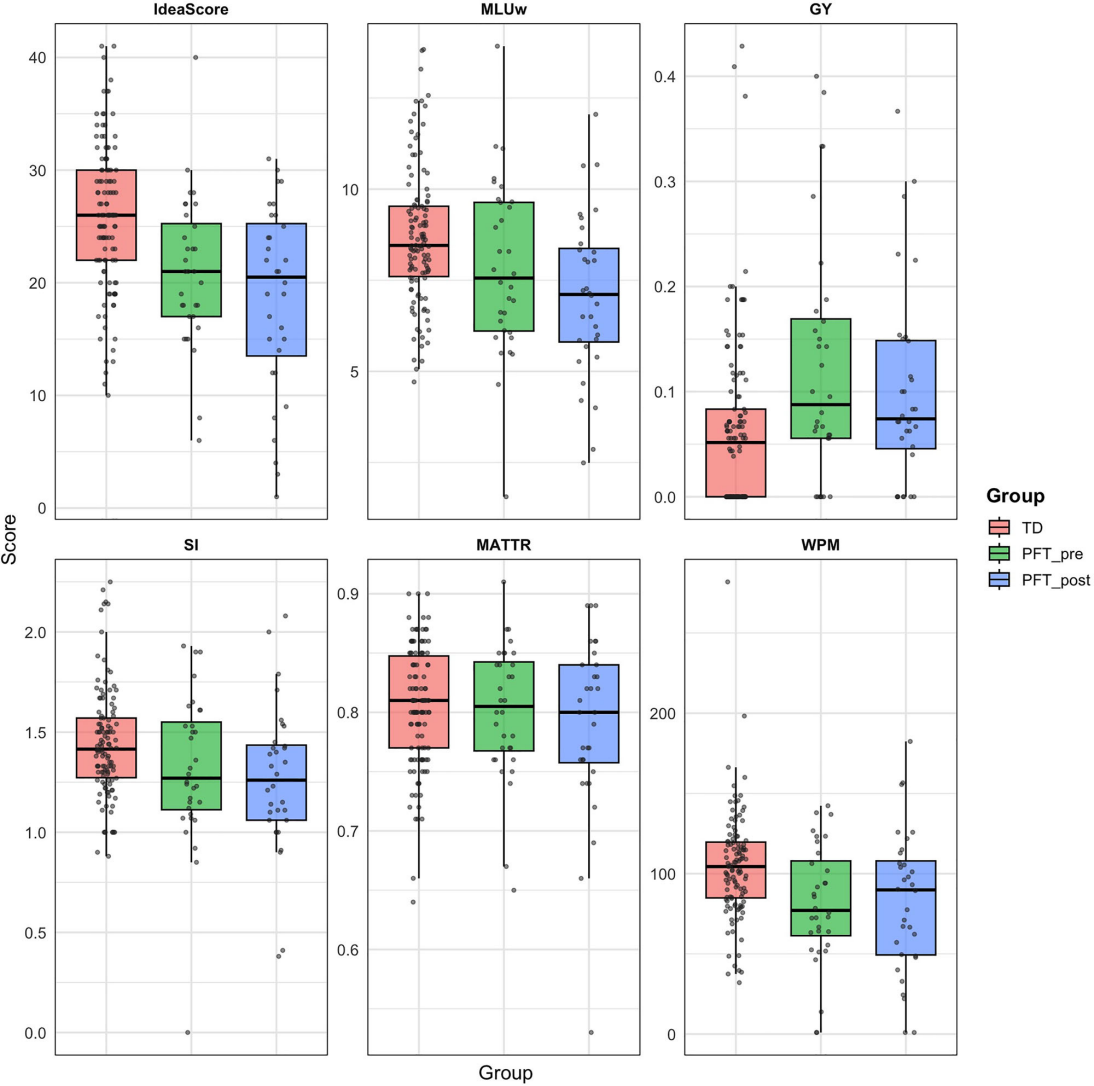
Boxplots comparing narrative macrostructural (ideascore) and microstructural (MLUw, GY, SI, MATTR, WPM) outcomes between TD children and children with PFT assessed pre‐ and postoperatively. Abbreviations: Ideascore = narrative macrostructure score; GY = grammaticality; MATTR = moving average type–token ratio; MLUw = mean length of utterance in words; SI = subordination index; WPM = words per minute.

A visualisation of these group differences is provided in Figure 2. The boxplots show the distribution of scores for ideascore and all microstructural measures (MLUw, GY, SI, MATTR, WPM), complementing the model estimates. For GY, higher values indicate a greater proportion of grammatical errors.

**FIGURE 3:**
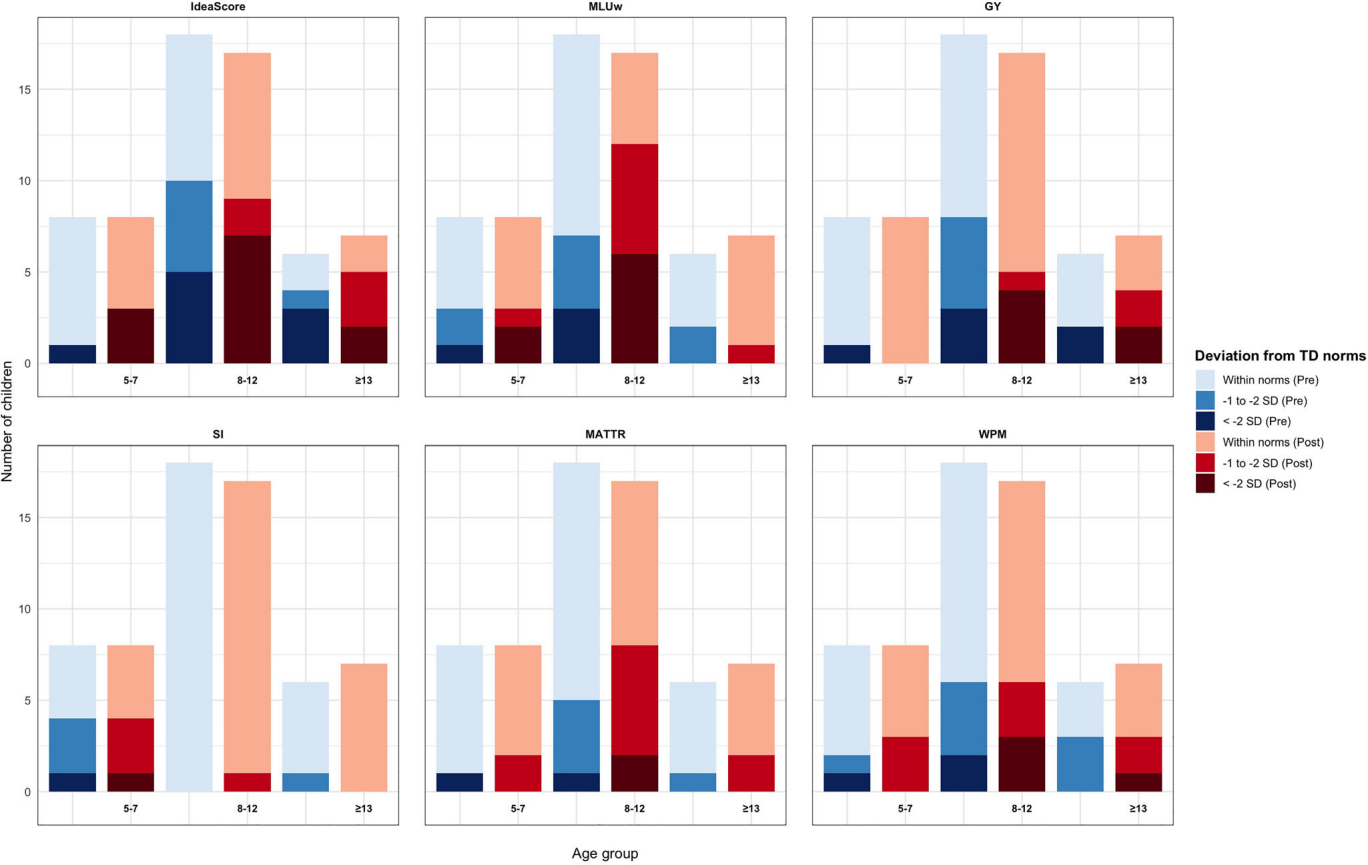
Number of children with PFT classified in relation to the TD mean (within one SD, 1–2 SD below, or more than 2 SD below), shown separately for pre‐ and postoperative assessments and across age groups (5–7, 8–12, ≥13 years). For GY, higher values reflect poorer accuracy, and deviations are above the TD mean (+1–2 SD, >+2 SD). Abbreviations: Ideascore = narrative macrostructure score; GY = grammaticality; MATTR = moving average type–token ratio; MLUw = mean length of utterance in words; SI = subordination index; WPM = words per minute.

To provide an indication of the clinical extent of difficulties we also explored the individual deviations from the TD mean pre‐ and postoperatively (Figure 3). For ideascore, deviations were already evident preoperatively, and these became more pronounced after surgery. The largest difficulties were observed among children aged 8–12 and ≥13 years, where several scored more than two SD below the TD mean. A similar pattern was found for MLUw, with deviations present before surgery and increasing afterwards, particularly in the 8–12 age group. For GY, most children in the youngest group (5–7 years) were within norms, while in the older groups a substantial proportion deviated, and several scored more than two SD above the TD mean both pre‐ and postoperatively. This deviation represents performance outside the range that includes approximately 95% of typically developing children. For WPM, difficulties were evident already preoperatively, and postoperative assessments showed a further increase in the number of children performing below the normative range, especially in the older age groups. For SI and MATTR, the majority of children in all age groups remained within the norms at both assessments, and only occasional deviations were observed.

### 3.2 Narrative Changes in Children With PFT

We then analysed whether the PFT group showed an average change from pre‐ to postoperative assessment. No statistically significant change was observed in either macro‐ or microstructure between pre‐ and postoperative assessment. For the macrostructural outcome ideascore, the scores declined from pre‐ to postoperative assessment, but the change was not statistically significant (*β* = −2.01, 95% CI −4.62 to 0.53, *p* = 0.133).

For the microstructural outcomes, no statistically significant changes were observed between pre‐ and postoperative assessments. MLUw showed a tendency towards shorter utterances postoperatively (*β* = −0.68, 95% CI −1.42 to 0.05, *p =* 0.079). There was an individual variation across both macro‐ and microstructural measures, where some children showed postoperative decline, others improved, and some remained relatively stable. Given this variability in postoperative outcomes, it is interesting to explore whether children who deteriorated were those with CMS or with motor speech disorder that is, postoperative dysarthria.

### 3.3 Narrative Changes in Relation to CMS and Dysarthria in Children With PFT

The group of children who developed postoperative CMS and for whom both preoperative and postoperative narratives were available was small, consisting of four children (one who had experienced mutism, three who had experienced reduced speech. For the macrostructural outcome ideascore, no significant change was found in relation to postoperative CMS, and none of the microstructural measures differed significantly between children with and without CMS (Table 3).

**TABLE 3:** Linear models of change in narrative macrostructural (ideascore) and microstructural (MLUw, GY, SI, MATTR, WPM) outcomes from pre‐ to postoperative assessment in relation to the presence of postoperative cerebellar mutism syndrome (CMS). The reference group consists of children without CMS. Models are adjusted for age and sex.

		B (95% CI)	p‐value	Standardised *β* (95% CI)
**Ideascore**	CMS	−6.09 (‐14.22–2.05)	0.132	−0.89 (‐2.07–0.30)
	Age	0.30 (‐0.89–1.49)	0.603	0.12 (‐0.37–0.61)
	Sex: boys	−4.62 (‐11.15–1.92)	0.154	−0.67 (‐1.62–0.28)
**MLUw**	CMS	−1.01 (‐3.63–1.62)	0.427	−0.50 (‐1.79–0.80)
	Age	0.11 (‐0.28–0.49)	0.569	0.15 (‐0.39–0.68)
	Sex: boys	−0.80 (‐2.91–1.31)	0.433	−0.39 (‐1.43–0.64)
**GY**	CMS	0.01 (‐0.15–0.17)	0.893	0.08 (‐1.24–1.40)
	Age	0.01 (‐0.01–0.04)	0.504	0.24 (‐0.30–0.78)
	Sex: boys	0.10 (‐0.05–0.25)	0.176	0.36 (‐0.70–1.43)
**SI**	CMS	−0.12 (‐0.57–0.33)	0.585	−0.34 (‐1.61–0.94)
	Age	0.03 (‐0.04–0.10)	0.346	0.24 (‐0.29–0.77)
	Sex: boys	−0.12 (‐0.49–0.24)	0.479	−0.35 (‐1.38–0.68)
**MATTR**	CMS	−0.06 (‐0.13–0.02)	0.089	−0.91 (‐1.98–0.15)
	Age	0.01 (0.00–0.02)	0.074	0.40 (‐0.04–0.84)
	Sex: boys	−0.02 (‐0.08–0.04)	0.453	−0.31 (‐1.17–0.55)
**WPM**	CMS	−38.73 (‐79.45–1.99)	0.061	−1.10 (‐2.26–0.06)
	Age	0.22 (‐5.73–6.18)	0.938	0.02 (‐0.46–0.49)
	Sex: boys	−26.34 (‐59.05–6.38)	0.107	−0.75 (‐1.68–0.18)

*Note*: Unstandardised (*β*) and standardised (*β*_std_) coefficients with 95% confidence intervals (CI) are reported.

Ideascore is narrative macrostructure score.

Abbreviations: CMS = Cerebellar Mutism Syndrome, MLUw = mean length of utterance in words; GY = grammaticality; SI = subordination index; MATTR = moving average type–token ratio; WPM = words per minute.

In relation to postoperative dysarthria, significant differences were observed for several outcomes. A significant decline was found in ideascore (*β* = −14.03, 95% CI −19.93 to −8.12, *p *< 0.001), MLUw (*β* = −3.11, 95% CI −5.50 to −0.71, *p *< 0.05), SI (*β* = −0.63, 95% CI −1.00 to −0.26, *p *< 0.01), MATTR (*β* = −0.09, 95% CI −0.16 to −0.03, *p *< 0.05), and WPM (*β* = −73.88, 95% CI −104.12 to −43.65, *p *< 0.001), while GY did not differ significantly between groups (Table 4).

**TABLE 4:** Linear models of change in narrative macrostructural (ideascore) and microstructural (MLUw, GY, SI, MATTR, WPM) outcomes from pre‐ to postoperative assessment in relation to the presence of postoperative dysarthria.

		B (95% CI)	p‐value	Standardised *β* (95% CI)
**Ideascore**	Postoperative dysarthria	−14.03 (−19.93 – −8.12)	**<0.001**	−2.04 (−2.90 – −1.18)
	Age	0.29 (−0.49–1.06)	0.443	0.12 (−0.20–0.44)
	Sex: male	−1.80 (−6.08–2.47)	0.385	−0.26 (−0.89–0.36)
**MLUw**	Postoperative dysarthria	−3.11 (−5.50 – −0.71)	**<0.05**	−1.53 (−2.70 – −0.35)
	Age	0.09 (−0.22–0.03)	0.553	0.12 (−0.31–0.56)
	Sex: male	−0.25 (−1.98–1.49)	0.766	−0.12 (−0.97–0.73)
**GY**	Postoperative dysarthria	0.05 (−0.13–0.22)	0.578	0.38 (−1.04–1.80)
	Age	0.01 (−0.02–0.03)	0.545	0.25 (−0.27–0.77)
	Sex: male	0.07 (−0.08–0.21)	0.334	0.31 (−0.72–1.33)
**SI**	Postoperative dysarthria	−0.63 (−1.00 – −0.26)	**<0.01**	−1.77 (−2.81 – −0.73)
	Age	0.02 (−0.02–0.07)	0.311	0.19 (−0.19–0.57)
	Sex: male	−0.03 (−0.05–0.05)	0.813	−0.09 (−0.84–0.67)
**MATTR**	Postoperative dysarthria	−0.09 (−0.16–0.03)	**<0.05**	−1.41 (−2.44 – −0.38)
	Age	0.01 (0.00–0.01)	**<0.05**	0.43 (0.05–0.81)
	Sex: male	0.00 (−0.05–0.05)	0.924	0.03 (−0.71–0.78)
**WPM**	Postoperative dysarthria	−73.88 (−104.12 – −43.65)	**<0.001**	−2.10 (−2.96 – −1.24)
	Age (years)	0.43 (−3.53–4.39)	0.820	0.03 (−0.28–0.35)
	Sex: male	−10.13 (−32.01–11.75)	0.341	−0.29 (−0.91–0.33)

*Note*: The reference group consists of children without dysarthria. Models are adjusted for age and sex. Unstandardised (*β*) and standardised (*β*_std_) coefficients with 95% confidence intervals (CI) are reported.

Ideascore = narrative macrostructure score; MLUw = mean length of utterance in words; GY = grammaticality; SI = subordination index; MATTR = moving average type–token ratio; WPM = words per minute.

**FIGURE 4:**
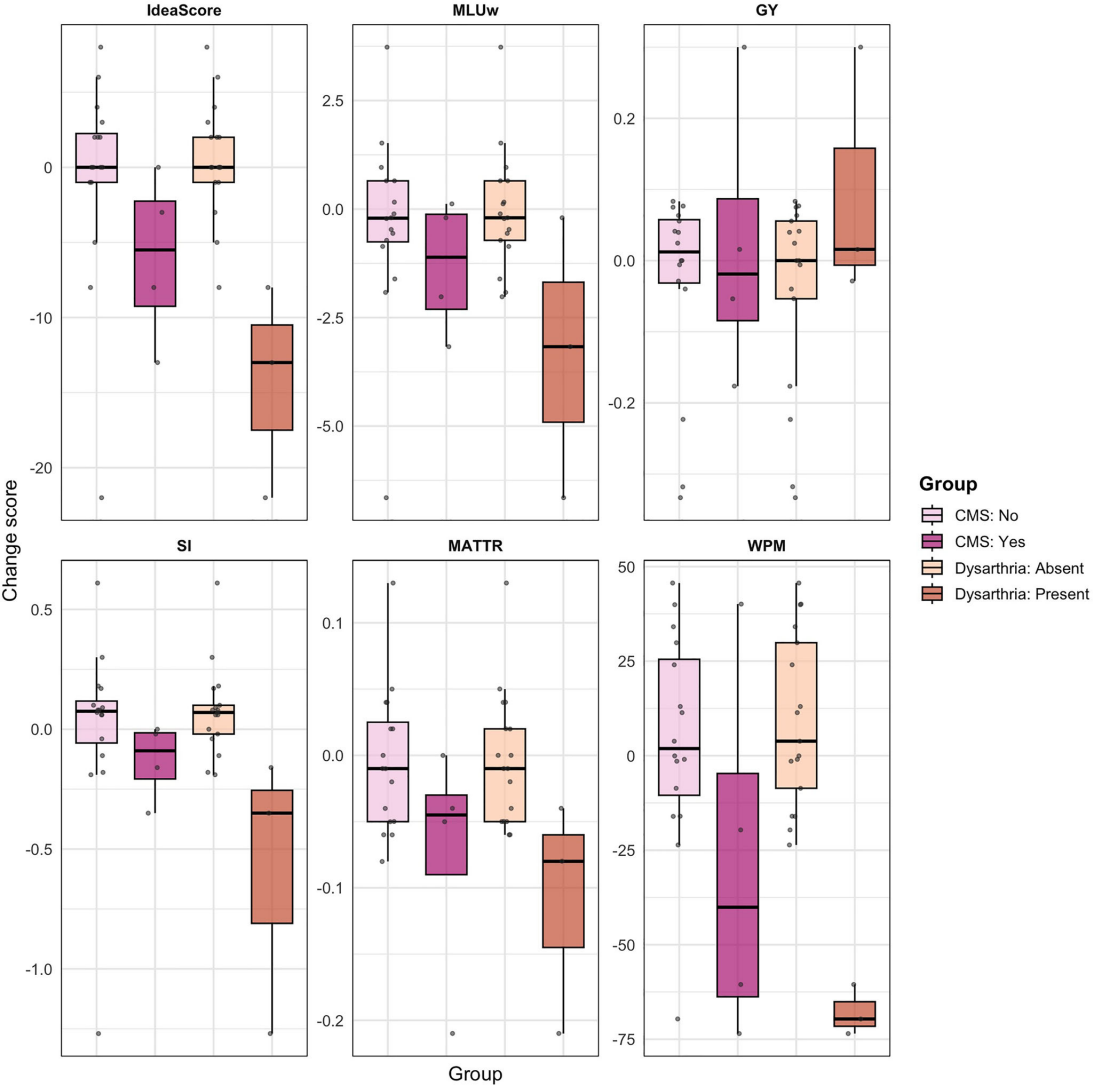
Individual change scores (post − pre) for macrostructural (ideascore) and microstructural (MLUw, GY, SI, MATTR, WPM) in relation to postoperative CMS and dysarthria. Abbreviations: Ideascore = narrative macrostructure score; GY = grammaticality; MATTR = moving average type–token ratio; MLUw = mean length of utterance in words; SI = subordination index; WPM = words per minute.

Individual change scores for CMS and for dysarthria across macro‐ and microstructural measures are shown in Figure 4.

## 4 Discussion

To our knowledge, this is the first study to investigate narrative ability in children with PFT at both the macrostructural and microstructural levels, including pre‐ and postoperative assessments. The findings provide valuable new insight into how narrative ability may be affected in the perioperative phase, and we first discuss the key difficulties identified across the macrostructural and microstructural levels. The second aspect we discuss concerns how these difficulties varied with age, as they were more pronounced in older children. Finally, we address the individual variation observed in postoperative outcome and note that children with CMS did not appear to experience a greater decline in narrative ability compared with others undergoing tumour surgery. Together, these findings deepen our understanding of the language consequences and variability in outcome following PFT surgery. They also highlight the clinical importance of conducting language assessments and follow‐up in children treated for PFT, in order to identify those in need of interventions.

### 4.1 Narrative Difficulties in Children With PFT Compared With TD Peers

The comparison with TD peers revealed narrative difficulties in children with PFT, affecting both the content and form of their narratives. The children with PFT performed significantly below TD peers on ideascore both pre‐ and postoperatively, showing difficulties with narrative macrostructure in the perioperative phase. This means that the children, for example, have failed to describe causal relationships between events and/or that essential components of the story are not included, resulting in narratives that are less complete and less informative compared with those of TD peers. This result aligns with earlier findings. Docking et al. (2016) reported lower macrostructural scores in children treated for brain tumours (including PFTs) compared with TD peers. However, in that study, the assessments were conducted several years after treatment, which for many children included chemotherapy and radiotherapy in addition to surgery, and the sample comprised both supratentorial and infratentorial tumours, however with no significant differences between these groups (Docking et al. 2016). To our knowledge, no previous study has investigated narrative macrostructural ability specifically in children with PFT, and our results showed that these difficulties were present both preoperatively and postoperatively and accordingly before additional treatment. These findings align with our previous reports of preoperative and postoperative word‐finding difficulties in children with PFT (Persson et al. 2023; Persson et al. 2025), indicating that the tumour itself may influence language abilities prior to surgery. Whether these difficulties arise from tumour‐related or surgical factors may not be of major clinical relevance in the perioperative phase, as children's communicative needs require similar interventions regardless of the underlying cause. However, understanding whether these difficulties are present before surgery is important for interpreting postoperative change, as it clarifies the relative contribution of tumour‐related and surgery‐related factors and helps relate such changes to the neurobiological mechanisms that may underlie the narrative difficulties observed in children undergoing treatment for PFT.

While difficulties in conveying narrative content were evident at the macrostructural level, additional differences were observed in language production at the microstructural level. In comparison with TD peers, the microstructural profile of children with PFT was characterised by shorter utterances, more errors in morphology and syntax, and reduced speech rate both pre‐ and postoperatively, while syntactic complexity and lexical diversity were preserved. This indicates that although children with PFT were able to produce complex sentences, their productions contained more morphological and syntactic errors than those of TD peers. Our results correspond with those reported by Svaldi et al. (2023), who found difficulties in MLU, grammatical accuracy, and fluency in children several years after cerebellar tumour surgery, suggesting that these problems are present both perioperatively and at long‐term follow‐up (Svaldi et al. 2023). However, in contrast to our findings, Svaldi et al. (2023) also reported deficits in lexical diversity in spontaneous language. In their study, with smaller sample size, lexical diversity was measured using type–token ratio, which is highly sensitive to sample length and has been shown to have limited validity as an indicator of lexical diversity in children's language samples (Ratner et al. 2024). We used MATTR instead, which is less affected by variation in sample length. The nature of the task is also likely to play a role, as the structured ERRNI narrative provides an external framework and fewer opportunities for lexical variation compared with spontaneous conversation.

Our results are in line with previous studies showing that children with DLD produce shorter utterances and more errors in morphology and syntax compared with TD peers (Delage and Frauenfelder 2020; Leonard 2014; Moscati et al. 2020). However, contrary to our results from children with PFT, children with DLD have been reported to show lower SI, suggesting reduced syntactic complexity (Araya et al. 2023; Delage and Frauenfelder 2020). Our findings indicate a specific language profile in this perioperative phase, characterised by difficulties in morphologic and syntactic accuracy, utterance length, and fluency, and a pattern that appears to differ from the difficulties observed in children with DLD.

Difficulties in both narrative form and content may affect children's communication, academic performance, and participation in social life (Berman 1988). Although the ideascore is derived from a structured, picture‐supported task, it reflects the child's ability to convey the main ideas of a story, an ability that is equally important in everyday communication.

### 4.2 Age‐Related Differences in Narrative Ability

The results further revealed an effect of age, with increasing narrative difficulties with increasing age in the children with PFT. Interestingly, the youngest children with PFT (5–7 years) largely performed within the normal range compared with age‐matched TD peers, both on the macrostructural measure ideascore and on several microstructural measures. In contrast, the largest deviations from TD norms were observed in the older age groups (8–12 and ≥13 years). This indicates that narrative difficulties, in both macro‐ and microstructure, were more pronounced among older children in our cohort. Similar age‐related patterns to those seen in our study have been reported in other paediatric groups (St John et al. 2019). In children with DLD, difficulties in narrative organisation and microstructure often become more evident with increasing linguistic and cognitive demands (Reilly et al. 2004; Isoaho et al. 2016; St John et al. 2019). Likewise, in children with acquired brain injury, language and communication difficulties often become more apparent as academic and communicative demands increase in school (MacDonald 2016). While our findings do not reflect developmental change per se, they suggest that the language differences between children with PFT and their TD peers become more pronounced as linguistic and cognitive demands increase with age. This pattern indicates that the age‐related differences observed in our study are in line with previous findings. One might not have expected the Fish Story task to be sufficiently demanding to reveal such differences, as for the older children it represents a relatively low‐demand task. More complex discourse contexts, such as expository or argumentative tasks, have been shown to elicit more complex syntactic and morphological forms in older children and adolescents, as these tasks place higher demands on language planning and reasoning (Berman and Verhoeven 2002; Nippold et al. 2008, 2007, 2014, 2017), and may therefore serve as more sensitive tools for examining narrative ability in this age group. The fact that the difficulties were most evident among the older children, despite the simplicity of the task, suggests that the difficulties described here are substantial. These findings underline the importance of attending to both macro‐ and microstructural aspects of narrative ability in school‐age children treated for PFT. Narrative difficulties may be less obvious than, for example, motor‐speech problems, and risk being overlooked, yet they can have a marked effect in everyday life. Regular narrative follow‐up assessments over time may help identify difficulties that can become more apparent as linguistic and cognitive demands increase, allowing for timely intervention.

### 4.3 Postoperative Changes and Their Relations to CMS and Dysarthria

The substantial individual variation in narrative change profiles highlights the complexity of language outcomes after PFT and suggests that different factors may underlie postoperative trajectories. Our findings reflect the heterogeneity in postoperative language outcomes, with some children improving and others declining, a pattern also noted by Di Rocco et al. (Di Rocco et al. 2010) and consistent with previous reports (Persson et al. 2025; Svaldi et al. 2023). Although the present study did not examine underlying factors for the interindividual variation in postoperative change profiles, our previous work has shown that a tumour in the fourth ventricle was significantly associated with poorer word‐finding abilities after surgery (Persson et al. 2025). A possible interpretation is that surgical removal of a tumour in this region may disrupt cerebello–cerebral networks important for language. However, language outcomes after posterior fossa tumour surgery are not uniform and likely reflect multiple interacting factors (Stoodley and Schmahmann 2010; Schmahmann et al. 2019). Among the factors that may contribute to this variation are postoperative CMS, known to result in language disorders (Catsman‐Berrevoets and Aarsen 2010; Svaldi et al. 2023), and dysarthria, which may secondarily affect speech production. Since we wanted to take preoperative level into account and therefore examined change, the analyses were based on a small subgroup of children with complete pre‐ and postoperative data. Children with postoperative CMS showed a general decline across both macrostructural and microstructural measures, although these changes did not reach statistical significance. The results may reflect the very small and heterogeneous CMS group, which included both children with total mutism and those with severely reduced speech. It is also important to note that, at the time of postoperative assessment (1–4 weeks after surgery), the children with CMS had recovered sufficiently to participate in the narrative task, which may indicate that they had a mild form of CMS. More prolonged or pronounced symptoms may be associated with greater subsequent language difficulties, and this should be considered when interpreting the findings for the CMS subgroup included in the present study. In our previous study of word‐finding ability, we found a tendency towards a clearer postoperative decline among children with mutism (Persson et al. 2025). Our results indicate that among these few individuals, CMS may influence narrative ability to varying degrees, in line with our previous findings suggesting that postoperative outcomes in children with PFT are best understood as existing on a continuum of severity rather than as discrete categories (Persson et al. 2025). A similar direction of change was observed for children with postoperative dysarthria, who also showed a decline across both macrostructural and microstructural measures, indicating a relation between motor speech disorder and narrative performance. Dysarthria is not a language disorder, and we interpret this relation as a reflection of the performance demands involved in narrative production. The relation is likely to reflect constraints related to articulation, coordination, and voice, which make narrative production more demanding and may limit utterance length, syntactic complexity, lexical diversity, and fluency. We consider dysarthria to be primarily related to the motor‐executive aspects of language production rather than to underlying language deficits, while acknowledging that the interaction between motor speech and language processes is complex. Regarding the microstructural measure WPM, it is closely related to motor speech disorders, as rate abnormalities are one of the main features of dysarthria (Duffy 2016). However, in the present study this measure captures speech rate as words per minute and incorporates pauses related to formulation processes (such as word‐finding and grammatical encoding), as well as revisions and self‐corrections during narrative production. Although WPM was significantly associated with postoperative dysarthria, the measure reflects both motor and language‐related factors. Given that only a small number of children in the present study were reported to have postoperative dysarthria, we interpret reduced speech rate as reflecting not only motor constraints but also language difficulties, noting that disentangling these contributions requires other methodological approaches. Our findings emphasise the individual variability in language outcomes after PFT, indicating that several factors may contribute to this variability. Dysarthria may contribute to narrative difficulties by placing additional constraints on speech production. Clinically, it is important to assess both narrative ability and motor speech in order to capture the different disorders that may underlie narrative difficulties after PFT. This is essential, as these difficulties require distinctly different forms of intervention.

## 5 Methodological Considerations

Several methodological aspects should be considered when interpreting the present findings. Although this is one of the largest studies including both pre‐ and postoperative narrative data in children with PFT, subgroup analyses, particularly those involving children with postoperative CMS or dysarthria, were based on small samples and should therefore be regarded as exploratory. The postoperative assessments were conducted 1–4 weeks after surgery, and variation in recovery and fatigue during this early phase may have influenced the results. Longer follow‐up periods are needed to determine whether the observed patterns persist or change over time. Follow‐up studies would also allow investigation of longer‐term outcomes in children with more severe or prolonged CMS who were not represented at the short‐term postoperative assessment in the present study. The structured ERRNI Fish Story task allowed for standardised data collection across centres and languages, which is a major strength in a multicentre study. However, the picture‐supported format may have constrained children's narrative production, especially regarding lexical diversity and complex syntax. While the task was clearly sensitive enough to capture difficulties among the older children in this study, it can be assumed that more demanding tasks might have revealed additional or different types of difficulties. Future studies could include elicitation tasks that place higher demands on language planning and reasoning, such as expository or argumentative contexts, to capture a broader range of language and communication abilities. Variability in test administration across professionals (e.g., speech‐language therapists, physicians, and nurses) cannot be excluded, although written instructions were followed in all settings.

## 6 Clinical Implications and Future Directions

The present findings underline the clinical importance of including narrative assessment in the evaluation of children treated for PFT. Assessing narrative ability provides valuable information about both the content and form of children's language, helping to identify those who may benefit from interventions. Because postoperative outcomes vary between individuals, speech and language follow‐up should be tailored to each child and include both language and motor‐speech assessments. Repeated assessment over time is also essential, as narrative difficulties may become more apparent when linguistic and cognitive demands increase with age and schooling.

Given the well‐documented association between narrative ability and academic achievement, particularly literacy and reading outcomes (Babayiğit et al. 2021; Griffin et al. 2004; Reese et al. 2011), future studies could link narrative outcomes in children with PFT to national educational register data to examine how narrative difficulties relate to school performance, including prior to tumour identification. Building on our previous work on pre‐ and postoperative language abilities in children with PFT and in those with CMS, future studies may also investigate how narrative abilities relate to the neurobiological underpinnings previously associated with language difficulties in these groups. Such work would allow for the development of more refined hypotheses about why some children experience narrative difficulties and how these difficulties evolve over time. A complementary line of enquiry concerns longitudinal follow‐up, examining how narrative and related language abilities change beyond the perioperative phase and whether recovery or continued vulnerability can be observed over time. Extending this work to larger and cross‐linguistic samples and combining narrative procedures with tasks that elicit more complex discourse, would make it possible to capture a broader range of developmental trajectories and deepen our understanding of how language difficulties evolve across childhood and adolescence. Such knowledge may also guide the development of future clinical approaches and intervention strategies aimed at supporting communication and participation in everyday life.

## 7 Conclusion

Children undergoing PFT surgery showed preoperative and postoperative difficulties in narrative macrostructure and microstructure compared with TD peers. These difficulties were most evident among older children, suggesting that narrative challenges become more apparent as linguistic and cognitive demands increase. Postoperative outcomes varied considerably between individuals; while we did not identify significant differences between children with and without CMS, children with postoperative dysarthria showed a decline across both narrative levels, interpreted as reflecting the performance demands involved in narrative production. Our findings emphasise the variability in language outcomes after PFT and highlight the importance of individualised follow‐up that includes assessments of both language and motor‐speech abilities.

## Funding

This study was supported by the Swedish Childhood Cancer Foundation, Queen Silvia's Jubilee Fund, Jonas Foundation and Independent Research Fund Denmark.

## Consent

Written informed consent was obtained from caregivers of all participants and by participants when appropriate.

## Conflicts of Interest

The authors declare no conflicts of interest.

## Ethics Statement

The study was approved by the Swedish Ethical Review Board (reference 2014/3:8) and (reference 2024‐05489‐01).

## Data Availability

Due to ethical considerations and data protection regulations, the datasets generated and analysed during the current study are not publicly available.

## References

[jlcd70248-bib-0001] Andreou, G. , and G Lemoni . 2025. “Narrative Skills of Children With Developmental Language Disorder: Retelling in Macrostructure.” Frontiers in Education 10: 1626433.

[jlcd70248-bib-0002] Araya, C. , C. J. Coloma , C. Quezada , and P Benavente . 2023. “Development of Clause Complexity in Children With Specific Language Impairment/Language Development Disorder: A Longitudinal Study.” Children 10, no. 7: 1152. 37508648 10.3390/children10071152 PMC10378421

[jlcd70248-bib-0003] Babayiğit, S. , S. Roulstone , and Y Wren . 2021. “Linguistic Comprehension and Narrative Skills Predict Reading Ability: A 9‐Year Longitudinal Study.” British Journal of Educational Psychology 91, no. 1: 148–168. 32432355 10.1111/bjep.12353

[jlcd70248-bib-0004] Berman, R. , and L Verhoeven . 2002. “Cross‐linguistic Perspectives on the Development of Text‐production Abilities: Speech and Writing.” Written Language & Literacy 5, no. 1: 1–43.

[jlcd70248-bib-0005] Berman, R. A 1988. “On the Ability to Relate Events in Narrative.” Discourse Processes 11, no. 4: 469–497.

[jlcd70248-bib-0006] Bishop, D. V. M 2004. Expression, Reception and Recall of Narrative Instrument: ERRNI Manual: Harcourt Assessment. University of Oxford.

[jlcd70248-bib-0007] Cámara, S. , M. C. Fournier , P. Cordero , et al. 2020. “Neuropsychological Profile in Children With Posterior Fossa Tumors With or Without Postoperative Cerebellar Mutism Syndrome (CMS).” Cerebellum 19, no. 1: 78–88. 31832994 10.1007/s12311-019-01088-4

[jlcd70248-bib-0008] Catsman‐Berrevoets, C. E. , and F. K Aarsen . 2010. “The Spectrum of Neurobehavioural Deficits in the Posterior Fossa Syndrome in Children After Cerebellar Tumour Surgery.” Cortex; A Journal Devoted to the Study of the Nervous System and Behavior 46, no. 7: 933–946. 20116053 10.1016/j.cortex.2009.10.007

[jlcd70248-bib-0009] Delage, H. , and U. H Frauenfelder . 2020. “Relationship Between Working Memory and Complex Syntax in Children With Developmental Language Disorder.” Journal of Child Language 47, no. 3: 600–632. 31775942 10.1017/S0305000919000722

[jlcd70248-bib-0010] De Smet, H. J. , H. Baillieux , C. Catsman‐Berrevoets , P. P. De Deyn , P. Marien , and P. F Paquier . 2007. “Postoperative Motor Speech Production in Children With the Syndrome of ‘Cerebellar’ Mutism and Subsequent Dysarthria: A Critical Review of the Literature.” European Journal of Paediatric Neurology 11, no. 4: 193–207. 17320435 10.1016/j.ejpn.2007.01.007

[jlcd70248-bib-0011] De Smet, H. J. , H. Baillieux , P. Wackenier , et al. 2009. “Long‐Term Cognitive Deficits Following Posterior Fossa Tumor Resection: A Neuropsychological and Functional Neuroimaging Follow‐Up Study.” Neuropsychology 23, no. 6: 694–704. 19899828 10.1037/a0016106

[jlcd70248-bib-0012] Di Rocco, C. , D. Chieffo , P. Frassanito , M. Caldarelli , L. Massimi , and G Tamburrini . 2011. “Heralding Cerebellar Mutism: Evidence for Pre‐Surgical Language Impairment as Primary Risk Factor in Posterior Fossa Surgery.” Cerebellum 10, no. 3: 551–562. 21476131 10.1007/s12311-011-0273-2

[jlcd70248-bib-0013] Di Rocco, C. , D. Chieffo , B. L. Pettorini , L. Massimi , M. Caldarelli , and G Tamburrini . 2010. “Preoperative and Postoperative Neurological, Neuropsychological and Behavioral Impairment in Children With Posterior Cranial Fossa Astrocytomas and Medulloblastomas: the Role of the Tumor and the Impact of the Surgical Treatment.” Childs Nervous System 26, no. 9: 1173–1188. 10.1007/s00381-010-1166-2 20552208

[jlcd70248-bib-0014] Docking, K. , N. Munro , T. Marshall , and L Togher . 2016. “Narrative Skills of Children Treated for Brain Tumours: The Impact of Tumour and Treatment Related Variables on Microstructure and Macrostructure.” Brain Injury 30, no. 8: 1005–1018. 27119976 10.3109/02699052.2016.1147602

[jlcd70248-bib-0015] Duffy, J. R 2016. “Functional Speech Disorders: Clinical Manifestations, Diagnosis, and Management.” In *Handb*ook of *Clin*ical *Neurol*ogy, vol. 139, edited by M. Hallet , J. Stone and A. Carson , 379–388. Elsevier. 10.1016/B978-0-12-801772-2.00033-3 27719858

[jlcd70248-bib-0016] Frizelle, P. , P. A. Thompson , D. McDonald , and D. V. M Bishop . 2018. “Growth in Syntactic Complexity Between Four Years and Adulthood: Evidence From a Narrative Task.” Journal of Child Language 45, no. 5: 1174–1197. 29860949 10.1017/S0305000918000144

[jlcd70248-bib-0017] Griffin, T. M. , L. Hemphill , L. Camp , and D. P Wolf . 2004. “Oral Discourse in the Preschool Years and Later Literacy Skills.” First Language 24, no. 2: 123–147.

[jlcd70248-bib-0018] Grønbæk, J. K. , D. Boeg Thomsen , K. Persson , R. Mathiasen , and M Juhler . 2023. “The Cerebellar Mutism Syndrome: Risk Assessment, Prevention and Treatment.” Advances and Technical Standards in Neurosurgery 46: 65–94. 37318570 10.1007/978-3-031-28202-7_4

[jlcd70248-bib-0019] Grønbæk, J. K. , M. Wibroe , S. Toescu , et al. 2021. “Postoperative Speech Impairment and Surgical Approach to Posterior Fossa Tumours in Children: A Prospective European Multicentre Cohort Study.” Lancet: Child & Adolescent Health 5, no. 11: 814–824. 34624241 10.1016/S2352-4642(21)00274-1

[jlcd70248-bib-0020] Gudrunardottir, T. , A. T. Morgan , A. L. Lux , et al. 2016. “Consensus Paper on Post‐Operative Pediatric Cerebellar Mutism Syndrome: The Iceland Delphi Results.” Childs Nervous System 32: 1195–1203. 10.1007/s00381-016-3093-3 27142103

[jlcd70248-bib-0021] Gustafsson, C. , E. Kristoffersson , and K Åkesson . 2009. ERRNI (Expression Reception and Recall of Narrative Instrument) En Utprövning och Utvärdering på svensktalande sexoch Åttaåringar Med Åldersadekvat Språklig Förmåga [Master's thesis]. Lund University.

[jlcd70248-bib-0022] Haberl, A. , J. Fleiß , D. Kowald , and S Thalmann . 2024. “Take the aTrain. Introducing an Interface for the **A**ccessible **Tr**anscription of **In**terviews.” Journal of Behavioral and Experimental Finance 41: 100891.

[jlcd70248-bib-0023] Isoaho, P. , T. Kauppila , and K Launonen . 2016. “Specific Language Impairment (SLI) and Reading Development in Early School Years.” Child Language Teaching and Therapy 32, no. 2: 147–157.

[jlcd70248-bib-0024] Kapantzoglou, M. , G. Fergadiotis , and M. A Restrepo . 2017. “Language Sample Analysis and Elicitation Technique Effects in Bilingual Children With and Without Language Impairment.” Journal of Speech, Language, and Hearing Research 60, no. 10: 2852–2864. 10.1044/2017_JSLHR-L-16-0335 28915297

[jlcd70248-bib-0025] Kupersmitt, J. R. , and E Nicoladis . 2021. “A Developmental Study of Expressing Simultaneous Events in Film‐Based Narratives.” First Language 41, no. 6: 708–736.

[jlcd70248-bib-0026] Leonard, L. B 2014. “Specific Language Impairment Across Languages.” Speech and Language Impairments in Children 8: 115–129. 10.1111/cdep.12053 PMC3994122 24765105

[jlcd70248-bib-0027] MacDonald, S 2016. “Assessment of Higher Level Cognitive‐communication Functions in Adolescents With ABI: Standardization of the Student Version of the Functional Assessment of Verbal Reasoning and Executive Strategies (S‐FAVRES).” Brain Injury 30, no. 3: 295–310. 26679634 10.3109/02699052.2015.1091947

[jlcd70248-bib-0028] Malvern, D. , B. Richards , N. Chipere , and P Durán . 2004. Lexical Diversity and Language Development. Springer.

[jlcd70248-bib-0029] Miller, J. F. , K. Andriacchi , A. Nockerts , M. F. Westerveld , and G Gillon . 2016.Assessing Language Production Using SALT Software: A Clinician's Guide to Language Sample Analysis. SALT Software.

[jlcd70248-bib-0030] Moscati, V. , L. Rizzi , I. Vottari , A. M. Chilosi , R. Salvadorini , and M. T Guasti . 2020. “Morphosyntactic Weaknesses in Developmental Language Disorder: The Role of Structure and Agreement Configurations.” Journal of Child Language 47, no. 5: 909–944. 31957622 10.1017/S0305000919000709

[jlcd70248-bib-0031] Nippold, M. A. , M. W. Frantz‐Kaspar , P. M. Cramond , C. Kirk , C. Hayward‐Mayhew , and M MacKinnon . 2014. “Conversational and Narrative Speaking in Adolescents: Examining the Use of Complex Syntax.” Journal of Speech, Language, and Hearing Research 57, no. 3: 876–886. 10.1044/1092-4388(2013/13-0097) 24167229

[jlcd70248-bib-0032] Nippold, M. A. , M. W. Frantz‐Kaspar , and L. M Vigeland . 2017. “Spoken Language Production in Young Adults: Examining Syntactic Complexity.” Journal of Speech, Language, and Hearing Research 60, no. 5: 1339–1347. 10.1044/2016_JSLHR-L-16-0124 28492843

[jlcd70248-bib-0033] Nippold, M. A. , T. C. Mansfield , and J. L Billow . 2007. “Peer Conflict Explanations in Children, Adolescents, and Adults: Examining the Development of Complex Syntax.” American Journal of Speech‐Language Pathology 16, no. 2: 179–188. 17456896 10.1044/1058-0360(2007/022)

[jlcd70248-bib-0034] Nippold, M. A. , T. C. Mansfield , J. L. Billow , and J. B Tomblin . 2008. “Expository Discourse in Adolescents With Language Impairments: Examining Syntactic Development.” American Journal of Speech‐Language Pathology 17, no. 4: 356–366. 18840698 10.1044/1058-0360(2008/07-0049)

[jlcd70248-bib-0035] Ostrom, Q. T. , M. Price , C. Neff , et al. 2023. “CBTRUS Statistical Report: Primary Brain and Other Central Nervous System Tumors Diagnosed in the United States in 2016–2020.” Neuro‐Oncology 25, no. 12 S2: iv1–iv99. 37793125 10.1093/neuonc/noad149 PMC10550277

[jlcd70248-bib-0036] Partanen, L. , N. Korkalainen , K. Mäkikallio , P. Olsén , H. Heikkinen , and A Yliherva . 2020. “Foetal Growth Restriction Has Negative Influence on Narrative Skills in 8‐10‐year‐old Children.” Acta Paediatrica 109, no. 8: 1595–1602. 31869483 10.1111/apa.15146

[jlcd70248-bib-0037] Persson, K. , D. Boeg Thomsen , Å. Fyrberg , et al. 2023. “Preoperative Word‐Finding Difficulties in Children With Posterior Fossa Tumours: A European Cross‐Sectional Study.” Child's Nervous System 40: 87–97. 10.1007/s00381-023-06119-4 PMC10761395 37682305

[jlcd70248-bib-0038] Persson, K. , J. Grønbæk , I. Tiberg , et al. 2025. “Postoperative Word‐Finding Difficulties in Children With Posterior Fossa Tumours: a Crosslinguistic European Cohort Study.” Childs Nervous System 41, no. 1: 128. 10.1007/s00381-025-06787-4 PMC11903548 40075014

[jlcd70248-bib-0039] Ratner, N. B. , Y. Han , and J. S Yang . 2024. “Should We Stop Using Lexical Diversity Measures in Children's Language Sample Analysis?” American Journal of Speech‐Language Pathology 33, no. 4: 1986–2001. 38838249 10.1044/2024_AJSLP-23-00457 PMC11253636

[jlcd70248-bib-0040] Reese, E. , A. Sparks , and S Suggate . 2011. “Assessing Children's Narratives.” In Research Methods in Child Language, edited by E. Hoff , 133–148. Wiley‐Blackwell.

[jlcd70248-bib-0041] Reilly, J. , M. Losh , U. Bellugi , and B Wulfeck . 2004. ““Frog, Where Are You?” Narratives in Children With Specific Language Impairment, Early Focal Brain Injury, and Williams Syndrome.” Brain and Language 88, no. 2: 229–247. 14965544 10.1016/S0093-934X(03)00101-9

[jlcd70248-bib-0042] Riva, D. , and C Giorgi . 2000. “The Cerebellum Contributes to Higher Functions During Development: Evidence From a Series of Children Surgically Treated for Posterior Fossa Tumours.” Brain 123, no. Pt 5: 1051–1061. 10775549 10.1093/brain/123.5.1051

[jlcd70248-bib-0043] Schmahmann, J. D. , X. Guell , C. J. Stoodley , and M. A Halko . 2019. “The Theory and Neuroscience of Cerebellar Cognition.” Annual Review of Neuroscience 42, no. 1: 337–364. 10.1146/annurev-neuro-070918-050258 30939101

[jlcd70248-bib-0044] Semel, E. , E. H. Wiig , and W. A Secord . 2013. Clinical Evaluation of Language Fundamentals–Fifth Edition (CELF‐5): Examiner's Manual. Pearson.

[jlcd70248-bib-0045] St John, M. , C. Ponchard , O. van Reyk , et al. 2019. “Speech and Language in Children With Klinefelter Syndrome.” Journal of Communication Disorders 78: 84–96. 30822601 10.1016/j.jcomdis.2019.02.003

[jlcd70248-bib-0046] Stoodley, C. J. , and J. D Schmahmann . 2010. “Evidence for Topographic Organization in the Cerebellum of Motor Control Versus Cognitive and Affective Processing.” Cortex: A Journal Devoted to the Study of the Nervous System and Behavior 46, no. 7: 831–844. 20152963 10.1016/j.cortex.2009.11.008 PMC2873095

[jlcd70248-bib-0048] Svaldi, C. , P. Paquier , S. Keulen , et al. 2023. “Characterising the Long‐Term Language Impairments of Children Following Cerebellar Tumour Surgery by Extracting Psycholinguistic Properties From Spontaneous Language.” Cerebellum 23, no. 2: 523–544. 37184608 10.1007/s12311-023-01563-z PMC10951034

[jlcd70248-bib-0049] Van Mourik, M. , C. E. Catsman‐Berrevoets , E. Yousef‐Bak , P. F. Paquier , and H. R van Dongen . 1998. “Dysarthria in Children With Cerebellar or Brainstem Tumors.” Pediatric Neurology 18, no. 5: 411–414. 9650681 10.1016/s0887-8994(97)00232-4

[jlcd70248-bib-0050] Volden, J. , E. Dodd , K. Engel , et al. 2017. “Beyond Sentences: Using the Expression, Reception, and Recall of Narratives Instrument to Assess Communication in School‐Aged Children With Autism Spectrum Disorder.” Journal of Speech, Language, and Hearing Research 60, no. 8: 2228–2240. 10.1044/2017_JSLHR-L-16-0168 28785770

[jlcd70248-bib-0051] Wibroe, M. , J. Cappelen , C. Castor , et al. 2017. “Cerebellar Mutism Syndrome in Children With Brain Tumours of the Posterior Fossa.” BMC Cancer 17, no. 1: 439. 28637445 10.1186/s12885-017-3416-0 PMC5480181

[jlcd70248-bib-0052] Winters, K. L. , J. Jasso , J. E. Pustejovsky , and C. T Byrd . 2022. “Investigating Narrative Performance in Children With Developmental Language Disorder: A Systematic Review and Meta‐Analysis.” Journal of Speech, Language, and Hearing Research 65, no. 10: 3908–3929. 10.1044/2022_JSLHR-22-00017 36179252

